# Prevalence of strawberry allergy in Bosnian children and management

**DOI:** 10.1186/2045-7022-1-S1-P45

**Published:** 2011-08-12

**Authors:** Adnan Bajraktarevic, Slobodan Trninic, Semira Penava, Amra Mahinic, Begler Begovic, Amina Selmovic, Sabina Kurtagic, Teodora Frankic, Jasna Gutic, Amra Hujic, Lutvo Sporisevic

**Affiliations:** 1 Public Health Institution of Canton Sarajevo, Pediatrics Department, Sarajevo, Bosnia and Herzegovina; 2 Clinical Medical Center Sarajevo, Clinical Pharmacology, Sarajevo, Bosnia and Herzegovina; 3 Pediatrics Clinic Sarajevo, Department for allergology and pulmonology, Sarajevo, Bosnia and Herzegovina; 4 Pediatrics Clinic Sarajevo, Department for gastroenetrology, hepatology and immunology, Sarajevo, Bosnia and Herzegovina; 5 Pharmacy Faculty Sarajevo, Clinical Pharmacology, Sarajevo, Bosnia and Herzegovina; 6 General Hospital Sarajevo, Emergency Department, Sarajevo, Bosnia and Herzegovina; 7 Dermatologic Clinic Sarajevo, Allergology Department, Sarajevo, Bosnia and Herzegovina; 8 First Medical Aid New Sarajevo, Pediatrics Department, Sarajevo, Bosnia and Herzegovina

## Introduction

A strawberry allergy is an allergy to certain proteins found in strawberries . The specific symptoms that can vary considerably amongst children from a severe anaphylactic reaction to asthma , abdominal symptoms, eczema or headaches. Some experience an allergic reaction with itching and swelling in mouth and throat.

## Objective

The goals were to estimate the prevalence of strawberry food allergy and to describe trends in food allergy prevalence and health care use among Bosnian children.

## Methods and materials

The sample included 40 primary care pediatricians from Sarajevo during last ten years 2000-2010; 95% of the respondents reported providing care for strawberry allergic children patients. The specific criteria used to diagnose food allergy may therefore have a significant impact on the results of these studies, especially those used to measure the prevalence of strawberry allergy. The allergen was indentified using blood serum from children patients experiencing adverse reactions to strawberry.

## Results

Red strawberries cause allergies, but white ones do not. The symptoms for strawberry allergy occur after exposure to strawberry fruit and strawberry products. The prevalence of strawberry allergy peaks at 3% to 4% at two year of age and then falls progressively until late childhood, after which the prevalence remains stable at 0.5% to 1% in children from Bosnia and Herzegovina . Some cases of life-threatening conditions have been reported, such as anaphylactic reactions and asphixia due to the impossibility of breathing.

## Discussion

All children with strawberry food allergy should also be reevaluated by their allergist at regular intervals to determine whether the allergy has been outgrown.

## Conclusion

The best treatment consists of prevention: individuals should avoid eating any form of strawberries, including raw berries, jam, cakes, jellies and even some naturally-flavoured products. In most of the cases strawberry allergy is not a life threatening one. Being allergic to strawberries is fairly common specially in children.

